# *Raphidocelis subcapitata* (=*Pseudokirchneriella subcapitata*) provides an insight into genome evolution and environmental adaptations in the Sphaeropleales

**DOI:** 10.1038/s41598-018-26331-6

**Published:** 2018-05-23

**Authors:** Shigekatsu Suzuki, Haruyo Yamaguchi, Nobuyoshi Nakajima, Masanobu Kawachi

**Affiliations:** Center for Environmental Biology and Ecosystem Studies, National Institute for Environmental Studies, Ibaraki, Japan

## Abstract

The Sphaeropleales are a dominant group of green algae, which contain species important to freshwater ecosystems and those that have potential applied usages. In particular, *Raphidocelis subcapitata* is widely used worldwide for bioassays in toxicological risk assessments. However, there are few comparative genome analyses of the Sphaeropleales. To reveal genome evolution in the Sphaeropleales based on well-resolved phylogenetic relationships, nuclear, mitochondrial, and plastid genomes were sequenced in this study. The plastid genome provides insights into the phylogenetic relationships of *R*. *subcapitata*, which is located in the most basal lineage of the four species in the family Selenastraceae. The mitochondrial genome shows dynamic evolutionary histories with intron expansion in the Selenastraceae. The 51.2 Mbp nuclear genome of *R*. *subcapitata*, encoding 13,383 protein-coding genes, is more compact than the genome of its closely related oil-rich species, *Monoraphidium neglectum* (Selenastraceae), *Tetradesmus obliquus* (Scenedesmaceae), and *Chromochloris zofingiensis* (Chromochloridaceae); however, the four species share most of their genes. The Sphaeropleales possess a large number of genes for glycerolipid metabolism and sugar assimilation, which suggests that this order is capable of both heterotrophic and mixotrophic lifestyles in nature. Comparison of transporter genes suggests that the Sphaeropleales can adapt to different natural environmental conditions, such as salinity and low metal concentrations.

## Introduction

Chlorophyceae are genetically, morphologically, and ecologically diverse class of green algae^1^. The group is dominant, particularly in freshwater, and plays important roles in global ecosystems^2^. The Chlorophyceae are composed of five taxonomic orders: Sphaeropleales, Chlamydomonadales, Chaetophorales, Chaetopeltidales, and Oedogoniales^1^. The Sphaeropleales are a large group, and contain some of the most common freshwater species (e.g. *Scenedesmus*, *Desmodesmus*, *Tetradesmus*, and *Raphidocelis*)^3,4^, including some species used in applications such as bioassays and biofuel production. In particular, *Raphidocelis subcapitata* and *Desmodesmus subspicatus* are recommended for ecotoxicological bioassays by the Organization for Economic Cooperation and Development (OECD) (TG201, http://www.oecd.org/) because they have higher growth rates and greater sensitivity to various substances than other algae.

Genome evolution in the Sphaeropleales is little understood compared to that of the Chlamydomonadales. In the Chlamydomonadales, genomes of four species (*Chlamydomonas reinhardtii*, *C*. *eustigma*, *Gonium pectorale*, and *Volvox carteri* f. *nagariensis*) have been sequenced and analyzed thus far^5–8^. Comparative genome analyses have provided great insights into the evolution of green algae traits, such as flagella^5^, multicellularity^6,7^, and sexual reproduction^9^. In contrast, genome analyses of the Sphaeropleales are rare; only three genomes, that of *Monoraphidium neglectum*^10^, *Tetradesmus obliquus*^11^, and *Chromochloris zofingiensis*^12^, have been sequenced. Furthermore, their comparative analyses have not been performed. The comparative analyses should provide insights into genome evolution of the Sphaeropleales and adaptation to different freshwater environments. *M*. *neglectum* contains large amounts of lipids under a wide range of pH and salt conditions, and thus shows potential for lipid production^13^. Its genome is 68 Mbp and encodes 16,761 proteins, including many genes related to carbohydrate metabolism and fatty acid biosynthesis, and indicates that the vegetative cells have diploid characters. *T*. *obliquus*, which was formerly classified as *Acutodesmus obliquus* or *Scenedesmus obliquus*, is a model organism in the Sphaeropleales for biofuel production and organellar genetics. Its nuclear genome is 109 Mbp^11^, but its detailed genome structure (i.e. gene models and annotations) has not been described. Recently, the nuclear genome of *C*. *zofingiensis* (=*Chlorella zofingiensis*) has been sequenced; the genome size is 58 Mbp and it encodes 15,274 proteins. In the case of organellar genomes, the mitochondrial genome has unusual codon usages (i.e. UAG for leucine, not as a stop codon, and UCA as a stop codon, not for serine)^14,15^, and a split *cox2* (*cox2a*, and *cox2b*)^16^. The *cox2b* gene is located in the nuclear genome, and thus it appears to have been transferred to the nuclear genome via an endosymbiotic gene transfer (EGT)^17^. In the Chlamydomonadales, both *cox2a* and *cox2b* are in the nuclear genome; therefore, the genes of *T*. *obliquus* are thought to be an intermediate character in EGT^18^. These mitochondrial characters are conserved in the Sphaeropleales^17,19^. The plastid genomes of the Sphaeropleales have fewer structural variations than the Chlamydomonadales and the OCC clade (Oedogoniales, Chaetopeltidales, and Chaetophorales)^20^. Therefore, the group is interesting for studying organellar and nuclear genome evolution in green algae.

*R*. *subcapitata* NIES-35 was formerly known as *Pseudokirchneriella subcapitata* or ‘*Selenastrum capricornutum*’^4^. On the basis of 18S rRNA phylogeny, *R*. *subcapitata* belongs to the family Selenastraceae, order Sphaeropleales, but its phylogenetic position in the group has not been resolved^4,21,22^. Although well-resolved phylogenetic relationships have been recently reported for the Sphaeropleales, using plastid or mitochondrial genome-encoded proteins^19,20,23,24^, the organellar genomes of *R*. *subcapitata* have not been sequenced. In this study, we sequenced the nuclear, mitochondrial, and plastid genomes of *R*. *subcapitata*, and compared them to other Sphaeropleales species genomes to reveal genome evolution in the order based on well-resolved phylogenetic relationships and to understand their genetic background in relation to high sensitivity to chemicals (e.g. metals). The plastid and mitochondrial genomes provide insights into the phylogenetic relationships of *R*. *subcapitata* and complex evolutionary histories in the order Sphaeropleales. The nuclear genome of *R*. *subcapitata* is the most compact in this order, and comparison of proteins indicates that the Sphaeropleales can adapt to a variety of nutrient and environmental conditions.

## Results and Discussion

### Phylogenetic analyses

**Figure 1:**
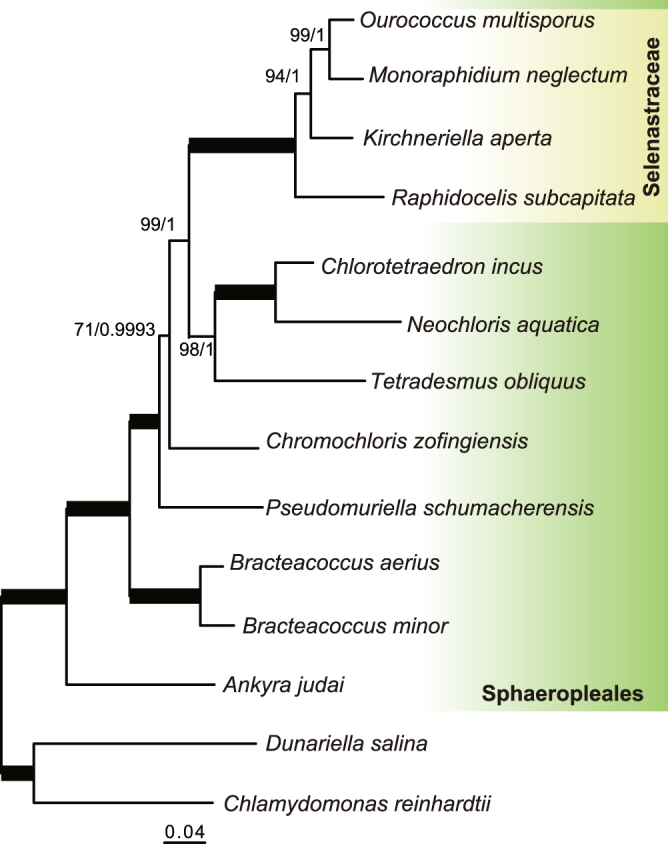
ML phylogenetic tree of the Sphaeropleales using 55 plastid-encoded proteins. The best tree was reconstructed using a concatenated dataset of 11,649 amino acids. Values at the nodes represent bootstrap supports (BP) of 200 replicates (right) and Bayesian posterior probabilities (BPP) (left). BP < 50 or BPP < 0.9 are not shown. Bold lines represent BP = 100 and BPP = 1.00.

To infer the phylogenetic position of *R*. *subcapitata* within the Sphaeropleales, we performed phylogenetic analyses using two datasets: 55 plastid-encoded or 13 mitochondrion-encoded proteins (Fig. 1; Supplementary Fig. S1). In both trees, *R*. *subcapitata* formed a monophyletic group with the other Selenastraceae species, *M*. *neglectum*, *Ourococcus multisporus*, and *Kirchneriella aperta*. There was robust support (BP = 100 and BPP = 1.00) for inclusion of *R*. *subcapitata* in the Sphaeropleales; however, the topologies of the trees were different. The tree using plastid-encoded proteins resolves the phylogenetic relationships better than the mitochondrial tree because it had higher supporting values (BPs at all nodes ≥70) and was based on more amino acids than that based on mitochondrion-encoded proteins. In the plastid-based tree, *R*. *subcapitata* was the most basal lineage in the Selenastraceae (BP = 94 and BPP = 1.00) (Fig. 1). *M*. *neglectum* and *O*. *multisporus* were sister species showing robust support (BP = 99 and BPP = 1.00). The Selenastraceae was a sister group to *T*. *obliquus*, *Neochloris aquatica*, and *Chlorotetraedron incus* (BP = 99, BPP = 1.00). *Chromochloris zofingiensis* was monophyletic with the Selenastraceae, *T*. *obliquus*, *N*. *aquatica*, and *C*. *incus*, with moderate support (BP = 71, BPP = 0.9993). Therefore, *C*. *zofingiensis* was probably the most basal among the four species with available nuclear genomes of the Sphaeropleales.

### Evolution of plastid and mitochondrial genomes

**Figure 2:**
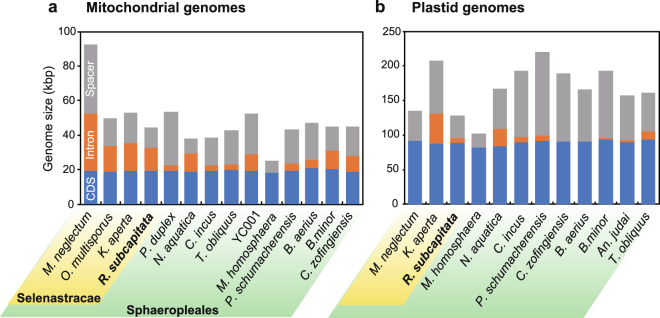
Size distributions of organellar genomes of *Raphidocelis subcapitata* and other Sphaeropleales species. (**a**) Mitochondrial genome of *R*. *subcapitata*. (**b**) Plastid genome of *R*. *subcapitata*. Blue, orange, and grey bars show the total sizes of coding sequences (CDSs), introns, and spacer regions, respectively.

In the Sphaeropleales, 17 mitochondrial and 11 plastid genomes have been submitted so far to comparative analyses^10,15,19,20,23–26^. We sequenced the complete mitochondrial and plastid genomes of *R*. *subcapitata* and compared these sequences to those of other Sphaeropleales to reveal organellar genome evolution of the order, and particularly of the family Selenastraceae. The mitochondrial genome of *R*. *subcapitata* was circular and 44,268 bp in size (Supplementary Fig. S2a); this genome contained a large tandem repeat region with a 10-mer unit repeated at least 11 times at position 21,665. Protein-coding genes could be translated following the genetic code of the mitochondria of *T*. *obliquus*^14^. There were 13 conserved protein-coding genes (3 cytochrome oxygenases, 1 cytochrome b, 7 NADH dehydrogenase subunits, and 2 ATP synthase subunits), 6 fragmented rRNAs, and 28 tRNAs; as observed for other Sphaeropleales species, 16S rRNA and 23S rRNA were separated into two and four fragments, respectively (Supplementary Table S1). Cox2 was split and its N-terminus (Cox2a) was encoded in the mitochondrial genome, similar to other mitochondrial genomes. Sphaeropleales species encode the C-terminus of Cox2 (Cox2b) in the nuclear genome^17^, and this was indeed the case in *R*. *subcapitata* (see below). *R*. *subcapitata* had 11 introns in *cox1*, *cob*, and *rrl4*, and 11 intronic open reading frames (ORFs), possibly encoding LAGLIDADG or GIY-YIG endonucleases. Except for an intron in *rrl4*, the introns of *R*. *subcapitata* were inserted at the same positions as that of at least one other Sphaeropleales species (Supplementary Fig. S3a–c), suggesting that they had the same origin. Selenastraceae species contained larger amounts of intronic sequences in the mitochondrial genomes than other Sphaeropleales species (Fig. 2a, Supplementary Fig. S3a–c), suggesting that there was a gain in introns in a common ancestor of the Selenastraceae. ProgressiveMauve analysis^27^ was performed to reveal the genome arrangements in the Selenastraceae. The gene order in the mitochondrial genome of *R*. *subcapitata* was identical to those of *K*. *aperta*, *O*. *multisporus*, and *M*. *neglectum*; however, there was a divergent region in an intron of *cob* (Supplementary Fig. S4). Overall, *R*. *subcapitata*, *K*. *aperta*, and *O*. *multisporus* had genome structures with high similarities, although *K*. *aperta* possessed longer introns in *rrl2* and *rrl4*. In contrast, *M*. *neglectum* had larger intergenic regions and introns (Fig. 2a; Supplementary Fig. S4), which may have been caused by a secondary genome size expansion in this species.

The plastid genome of *R*. *subcapitata* was circularly mapped and 128,080 bp in size, including two 18,858 bp inverted repeats (IRs) (Supplementary Fig. S2b). Sixty-nine conserved protein-coding genes, 6 rRNAs, 30 tRNAs, and 7 intronic ORFs were found in the plastid genome (Supplementary Table S2). The IR had 1 *psbA*, 3 rRNAs, and 3 tRNAs. Two introns were inserted in *psaA* and 4 introns in 2 copies of *rrl*, all of which possessed an intronic ORF encoding LAGLIDADG endonuclease. Exceptionally, there was an ORF encoding maturase in an intergenic region between *psbE* and *psaA-1*; *psaA* was split into three fragments, which were dispersed in the genome, suggesting that they could be transcribed with *trans-*splicing, as in *T*. *obliquus*^26^. Among the Sphaeropleales species studied, *M*. *homosphaera* contained the smallest plastid genome (102.7 kbp), and *R*. *subcapitata* the second smallest, mainly because of small intergenic regions (Fig. 2b). Because the phylogenetic relationships between *R*. *subcapitata* and *M*. *homosphaera* are unclear, we cannot determine whether the genome reduction is derived. Of the three Selenastraceae species, *R*. *subcapitata* and *K*. *aperta* showed a completely conserved gene set (Supplementary Table S2), with *K*. *aperta* possessing a larger number of introns and intergenic regions (Fig. 2b). *M*. *neglectum* had an additional *atpF* in the IRs, one of which was fragmented; thus, it may be a pseudogene. Based on phylogenetic relationships, IRs increased in length in *M*. *neglectum* after branching in the Selenastraceae. The gene orders of *M*. *neglectum* and *K*. *aperta* were identical, but *R*. *subcapitata* contained an inversion of *psbK*–*psbC* (Supplementary Fig. S5), suggesting that this inversion occurred independently.

### General features of the nuclear genome of *R*. *subcapitata*

We sequenced 4.1 Gbp reads using Illumina MiSeq. Based on assembly and scaffolding, we obtained 417 scaffolds (≥1 kbp). Major scaffolds (≥5 kb) were 51,162,697 bp in total (300 scaffolds) (Table 1). The assembly size was similar to the estimated genome size (46,790,751 bp), based on a k-mer analysis (Supplementary Fig. S6). To confirm the completeness of the genome, we performed BUSCO analysis, which assesses genome completeness to find the Benchmarking Universal Single-Copy Orthologs (BUSCOs) in a target genome^28^. The analysis was based on the eukaryote dataset, and showed 91.7% complete BUSCOs, which was higher than those of *C*. *zofingiensis*, *M*. *neglectum*, and *T*. *obliquus* (84.5%, 58.5%, and 79.9%, respectively) (Supplementary Table S3). Additionally, 95.8% and 95.6% of the RNA-seq reads were mapped into ≥1 kb and ≥5 kb scaffolds, respectively. Therefore, we obtained a nearly complete genome for *R*. *subcapitata*. The 300 major scaffolds were annotated using the funannotate pipeline based on *ab initio* and transcripts-based gene prediction. Repeat sequences (12.45% of the major scaffolds) were masked, and most (10.5% of the major scaffolds) were simple repeats. At least 5 rRNAs, 46 tRNAs, and 13,383 protein-coding genes were predicted. Additionally, 11,902 protein-coding genes (88.9% of the prediction) were detected by RNA-seq. The remaining 11.1% of gene models were not mapped with the RNA-seq reads; therefore, they may be rarely expressed genes and some may be pseudogenes. The protein-coding genes contained 76,178 introns at a density of 5.7 introns per gene.

**Table 1:** Statistical comparison of nuclear genomes of *R. subcapitata, M. neglectum, T. obliquus, C. zofingiensis* and *C. reinhardtii*.

	*Raphidocelis subcapitata*	*Monoraphidium neglectum*	*Tetradesmus obliquus*	*Chromochloris zofingiensis* ^**^	*Chlamydomonas reinhardtii* ^***^
Strain	NIES-35	SAG 48.87	UTEX393	SAG 211-14	CC503
Reference	This study	Bogen *et al*. (2013)	Carreres *et al*. (2017), this study	Roth *et al*. (2017)	Merchant *et al*. (2008)
Assembly size (Mbp)	51 (≥5 kb)	68	108	58	111
Number of scaffolds	417 (≥1 kb), 300 (≥5 kb)	857 (>20 kb)	1,368	19 (chromosomes)	54
L50 (Scaffolds)	46	1,303	177	—	7
N50 (Scaffolds, kbp)	342	16	187	chromosomes	7,800
GC%	72	65	57	51	64
Number of CDSs	13,383	16,761	12,496	15,274	17,741
Mean protein length (aa)	561	348	452	427	736
Mean intron length (bp)	230	302	462	284	270
Number of introns per gene	5.7	4.0	7.8	4.0	7.5
coding%^*^	44.0	25.7	15.7	39^****^	35.2

^*^Only protein-coding genes.

^**^JGI v. 5.2.3.2.

^***^JGI v. 5.5.

^****^Roth *et al*. (2017).

**Figure 3:**
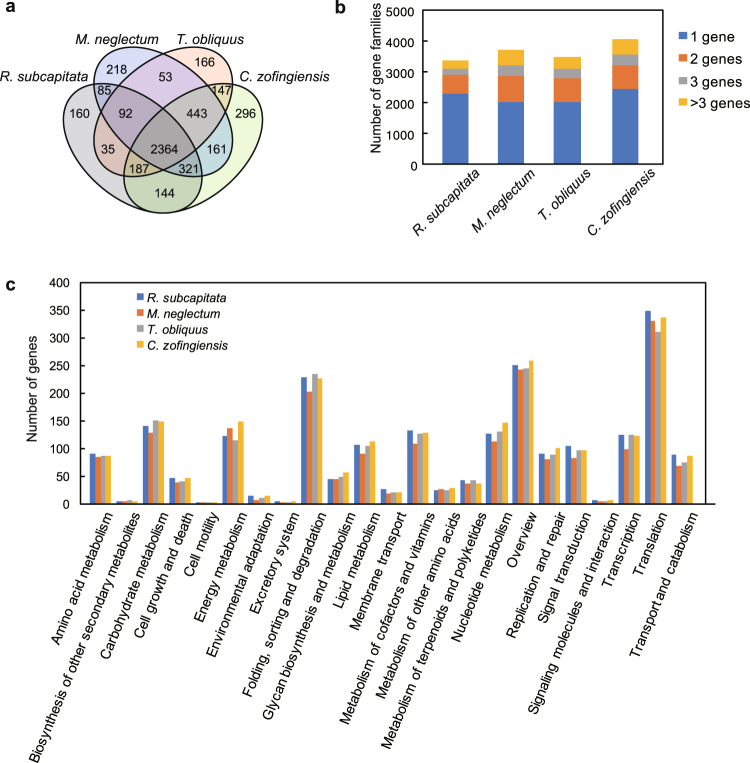
Comparison of gene families among *Raphidocelis subcapitata, Monoraphidium neglectum, Tetradesmus obliquus*, and *Chromochloris zofingiensis*. (**a**) Venn diagram of the gene families of *R*. *subcapitata*, *M*. *neglectum*, and *T*. *obliquus*. (**b**) The number and size of gene families. Gene families consisting of multiple genes are shown in red, grey, and orange according to their family size (two, three, and more than four, respectively). (**c**) Functional comparison of the genes according to KEGG classification.

The nuclear genome of *R*. *subcapitata* was smallest in the Sphaeropleales (Table 1). Thus far, the nuclear genomes of *M*. *neglectum*, *T*. *obliquus*, and *C*. *zofingiensis* have been sequenced^10–12^. The genomes differed greatly from that of *R*. *subcapitata* in size, gene number, coding capacity, and GC-content. *T*. *obliquus* has a 107.7 Mbp genome, which is nearly twice as large as that of *R*. *subcapitata*, but the two species possess a similar number of genes. The coding percentages of *R*. *subcapitata* and *T*. *obliquus* were 44.0% and 15.7%, respectively. These results suggest that the larger genome of *T*. *obliquus* is caused by a large amount of non-coding regions (i.e. intergenic regions and introns). The genomes of *M*. *neglectum* (68 Mbp) and *C*. *zofingiensis* (58 Mbp) were also smaller but with higher coding percentages (25.7% and 39%^12^, respectively) than that of *T*. *obliquus*. However, *M*. *neglectum* and *C*. *zofingiensis* possessed 16,761, and 15,274 genes, respectively, at least ~2,000 genes more than *R*. *subcapitata* and *T*. *obliquus*. We compared gene families, which were defined by TreeFam^29^, among *R*. *subcapitata*, *M*. *neglectum*, *T*. *obliquus*, and *C*. *zofingiensis*. Most of the families (58.2–69.8% of the total) were shared by them (Fig. 3a). *C*. *zofingiensis* had 4,063 gene families, ~300 more gene families than the others (Fig. 3b). However, only 296 unique gene families were found in *C*. *zofingiensis*, which was similar to that of the others (160–218 gene families) (Fig. 3a). Functional analyses using KEGG categories^30^ showed that most of the functions were shared in the Sphaeropleales (Fig. 3c). *M*. *neglectum* possessed the largest number of genes among the Sphaeropleales families considered in the study (Fig. 3b). Therefore, gene expansion in *M*. *neglectum* probably originated in the past as a complete or partial duplication of the genome. Bogen *et al.*^10^ reported that *M*. *neglectum* had a diploid character because their investigation of a ‘contig length vs. read count’ plot showed homozygous and heterozygous contigs. In this plot, contigs of homozygous loci have more than twice the read coverage than those of heterozygous loci^31^. However, such characters were not found in *R*. *subcapitata*, *T*. *obliquus* (Supplementary Fig. S7a,b), and *C*. *zofingiensis*^12^. Therefore, we consider that vegetative cells of the Sphaeropleales are basically haploid. The haploid character, and the small size and simplicity of the *R*. *subcapitata* genome are of great advantage for transformation and genome editing. Interestingly, GC-content of *R*. *subcapitata* was 71.6%, which was higher than that of *M*. *neglectum*, *T*. *obliquus*, and *C*. *zofingiensis* (Table 1). Roth *et al.*^12^ indicated that the high GC-content of several algae was associated with more fragmentary assemblies. However, the *R*. *subcapitata* genome had higher GC-content than *M*. *neglectum* and *T*. *obliquus* despite the less fragmentary assembly of *R*. *subcapitata* (Table 1; Supplementary Table S3). Therefore, the genome of *R*. *subcapitata* might have high GC-content in nature. Codon usage of *R*. *subcapitata* was highly biased; we found that although all codons were used, there was a greater proportion of GC at synonymous codons, e.g. cysteine was coded for by 20% UGU and 80% UGC codons (Supplementary Table S4). Additionally, the GC-content of transcripts (coding regions) in *R*. *subcapitata* was 74.6%, which was higher than that of spacer regions (66.2%). These results suggest that the GC bias is related to the high gene density and coding length of *R*. *subcapitata*. In mammalian species, it is known that high GC-content is positively correlated to gene density^32^ and coding length^33^.

### Restricted endosymbiotic gene transfer of mitochondrial genes in the Sphaeropleales

Species in the Sphaeropleales have split *cox2* (*cox2a* and *cox2b*) genes, with *cox2a* located in the mitochondrial genome and *cox2b* located in the nuclear genome, based on the difference in codon usage between the mitochondria and nucleus and the presence of spliceosomal introns. Because the Chlamydomonadales have both split *cox2* genes in their nuclear genome^16^, the Sphaeropleales appear to show an intermediate trait of gene migration to the nucleus^17^.

To gain further insights into EGT from the mitochondrial genome in the Sphaeropleales, we searched for *cox2b* and nuclear mitochondrial DNA segments (NUMTs), which are nuclear sequences that are similar to the mitochondrial genome sequences. We found a *cox2b* gene in *R*. *subcapitata*, *M*. *neglectum*, and *C*. *zofingiensis*. *T*. *obliquus* possessed two copies of *cox2b* with N terminal sequences that differed from those reported previously^17^. All of the *cox2b* possessed a ~50 aa N-terminal extension, which was very similar to that of the Chlamydomonadales (Supplementary Fig. S8), strongly supporting the conclusion that *cox2b* migrated to the nucleus in a common ancestor of the Chlamydomonadales and Sphaeropleales^17^.

**Figure 4:**
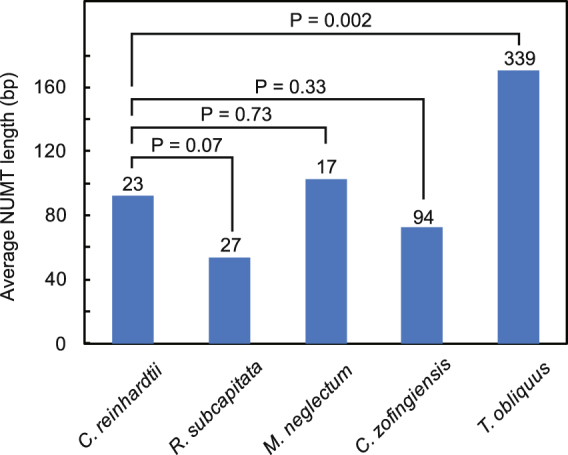
Comparison of NUMTs of *Raphidocelis subcapitata, Monoraphidium neglectum*, *Tetradesmus obliquus*, *Chromochloris zofingiensis*, and *Chlamydomonas reinhardtii*. Bars represent average length of NUMTs; the number of NUMTs are shown above the bars. P values (*t*-test) are shown.

We also performed homology searching of the mitochondrial sequences against the nuclear sequences. We did not find mitochondrial-encoded genes in nuclear protein-coding genes; however, we found some NUMTs in intergenic regions or introns of the nuclear genomes. *R*. *subcapitata* possessed 27 NUMTs with 30–119 bp with 88–100% similarity (Fig. 4; Supplementary Table S5), and *M*. *neglectum* possessed 17 NUMTs with 34–350 bp with 83–100% similarity. *C*. *zofingiensis* possessed 94 NUMTs, a greater number than in *R*. *subcapitata* and *M*. *neglectum*, with 31–279 bp with 82–100% similarity. These NUMT lengths were not significantly different from those in *C*. *reinhardtii* (P = 0.07–0.72, t-test) (Fig. 4). These results suggest that the Sphaeropleales and the Chlamydomonadales have similar amounts of ongoing mitochondrial DNA transfer to the nucleus. However, in the Sphaeropleales, *cox2a* has not been transferred to the nuclear genome; this might be caused by unusual codon usage in this order^17^. If the gene is transferred, it cannot be translated correctly to protein, and the DNA fragment may be eliminated immediately. Exceptionally, *T*. *obliquus* possessed many more NUMTs than the other Sphaeropleales, 339 NUMTs with 32–2,108 bp with 78–100% similarity (Supplementary Table S5), which were significantly larger in size than those of *C*. *reinhardtii* (P = 0.002, t-test) (Fig. 4). This may be explained by the lower gene density of *T*. *obliquus*. It had large spacer regions (3,647 bp on average) and introns (462 bp on average), therefore, it is likely that mitochondrial DNA fragments are transferred easily into the nucleus without disturbing genes.

### Conserved gene repertories for lipid metabolism in the Sphaeropleales

**Figure 5:**
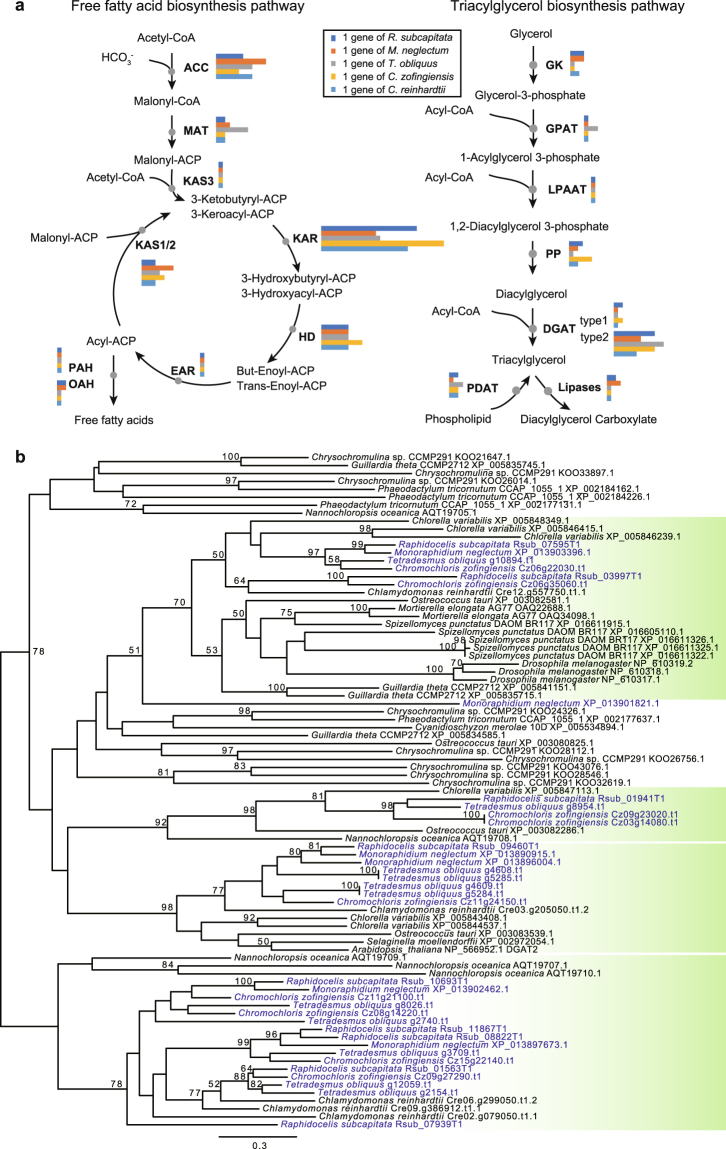
Pathways of free fatty acids and triacylglycerol in the Sphaeropleales. (**a**) Putative pathways and gene numbers of free fatty acids and triacylglycerol in the Sphaeropleales. The metabolic pathways were based on Bogen *et al.*^10^. Enzymes are shown in bold type. Bars represent the number of genes of *Raphidocelis subcapitata* (blue), *Monoraphidium neglectum* (red), *Tetradesmus obliquus* (grey), *Chromochloris zofingiensis* (orange), and *Chlamydomonas reinhardtii* (cyan). (**b**) Unrooted ML phylogenetic tree of DGAT2. Blue-coloured operational taxonomic units represent Sphaeropleales proteins. Green-coloured clades include the Sphaeropleales. Bootstrap support (BP) is indicated above the lines where BP is more than 50%. The ML analysis was performed using IQ-tree 1.5.5 with 215 amino acids with LG + F + G4 model. Non-parametric bootstrapping was performed 100 times. ACC: acetyl-CoA carboxylase; ACP: acyl carrier protein; MAT: acyl-carrier-protein S-malonyltranferase; KAS3: beta-ketoacyl-acyl carrier-protein synthase III; KAR: 3-oxoacyl-(acyl-carrier-protein) reductase; HD: hydrolyases; EAR: enoyl-acyl carrier protein reductase; KAS1/2: beta-ketoacyl-acyl-carrier-protein synthase I/II; PAH: palmitoyl-protein thioesterase; OAH: oleoyl-(acyl-carrier-protein) hydrolase; GK: glycerol kinase; GPAT: glycerol-3-phosphate O-acyltransferase; AGPAT: 1-acetylglycerol-3-phosphate O-acyltransferase; PP: phosphatidate phosphatase; DGAT: acyl-CoA:diacylglcerol acyltransferase; PDAT: phospholipid:diacylglycerol acyltransferase.

Some Sphaeropleales species are used for the production of biomass, particularly biofuels. *T*. *obliquus* and *M*. *neglectum* accumulate palmitate (C16:0) and oleate (C18:1) as major lipid constituents, which may be suitable feedstock for biodiesel production^10,34^. A congener of *M*. *neglectum*, *M*. *contortum*, can also be useful for biofuel production because of its robust growth, with efficient neutral lipid accumulation, and a favourable fatty acid profile under nitrogen starvation conditions^13^. *M*. *contortum* and *R*. *subcapitata* mainly accumulate palmitate and oleate^13,35^. We searched for genes related to lipid metabolism in the Sphaeropleales, based on metabolic pathways^10^, and compared them to those of *C*. *reinhardtii*. They shared all the genes except for enoyl-acyl-carrier-protein reductase (EAR) (Fig. 5a). The gene for EAR in *M*. *neglectum* could not be detected by our homology search; however, Bogen *et al.*^10^ reported the presence of EAR. The Sphaeropleales possessed more genes for acyl-CoA:diacylglycerol acyltransferase (DGAT) than *C*. *reinhardtii* (Fig. 5a). DGAT catalyses the last step in triacylglycerol (TAG) biosynthesis in the acyl-CoA dependent pathway, and is composed of two families with similar structure, DGAT type1 and DGAT type2 (also known as DGTT)^36^. Roth *et al.*^12^ discussed the difficulties for annotation of these genes because of their high sequence diversities, and 11 reported putative genes for DGAT type1 or 2. In our analyses, although the exact number of genes for DGAT with diacylglycerol acyltransferase activity could not be confidently determined, *R*. *subcapitata* and *T*. *obliquus* tentatively possessed 11 and 12 genes for DGAT, respectively, and most of them were homologs of DGAT type2 by our phylogenetic analysis. The DGAT type2 genes of the Sphaeropleales were present in four clades (Fig. 5b), and each clade included other green algae, suggesting that the increase in genes originated in gene duplication. An increase in DGAT genes is found in other oleaginous organisms, such as *Nannochloropsis* species^37,38^. In these species, the increase in DGAT type2 genes originated in their endosymbiont via EGT^37^, whereas the gene number increase in the Sphaeropleales was probably caused by gene duplication in their ancestor. For the other genes related to lipid biosynthesis, the Sphaeropleales possessed a large number of genes for ACC, KAR, and KAS1/2 (Fig. 5a) as mentioned in Bogen *et al*.^10^. These findings imply that the large number of DGAT genes may be related to high lipid productivity in the Sphaeropleales.

### Variation in nutrient transporter genes and environmental adaptation in the Sphaeropleales

The Sphaeropleales are known generally as a dominant group in freshwater and adapted to different environmental conditions e.g.^39,40^. Moreover, Sphaeropleales species have high sensitivity to exogenous substances^41^. To characterize the adaptive potential of the Sphaeropleales to environmental variability, we compared transporter genes among the Sphaeropleales and *C*. *reinhardtii*. Most of the transporter gene repertoires for nutrients and metals were similar among the Sphaeropleales and *C*. *reinhardtii* (Supplementary Table S6); however, the Sphaeropleales possessed markedly greater numbers of genes for H^+^/hexose transporters (2.A.1.1, TCAD ID), amino acid permeases (2.A.18.2), peptide transporters (2.A.17.3), aquaporin (1.A.8.8), and metal-nicotianamine transporters (2.A.67.2) than *C*. *reinhardtii* (Table 2).

**Table 2:** The number of genes for several transporters.

Annotation	TCAD ID	Number of genes				
		*R*. *subcapitata*	*M*. *neglectum*	*T*. *obliquus*	*C*. *zofingiensis*	*C*. *reinhardtii*
Aquaporin	1.A.8.8	3	3	8	4	1
Hexose transporter	2.A.1.1	13	14	12	12	3
Peptide transporter	2.A.17.3	5	8	3	3	1
Amino acid permease	2.A.18.2	8	7	11	4	0
Metal-nicotianamine transporter	2.A.67.2	6	4	5	4	0

**Figure 6:**
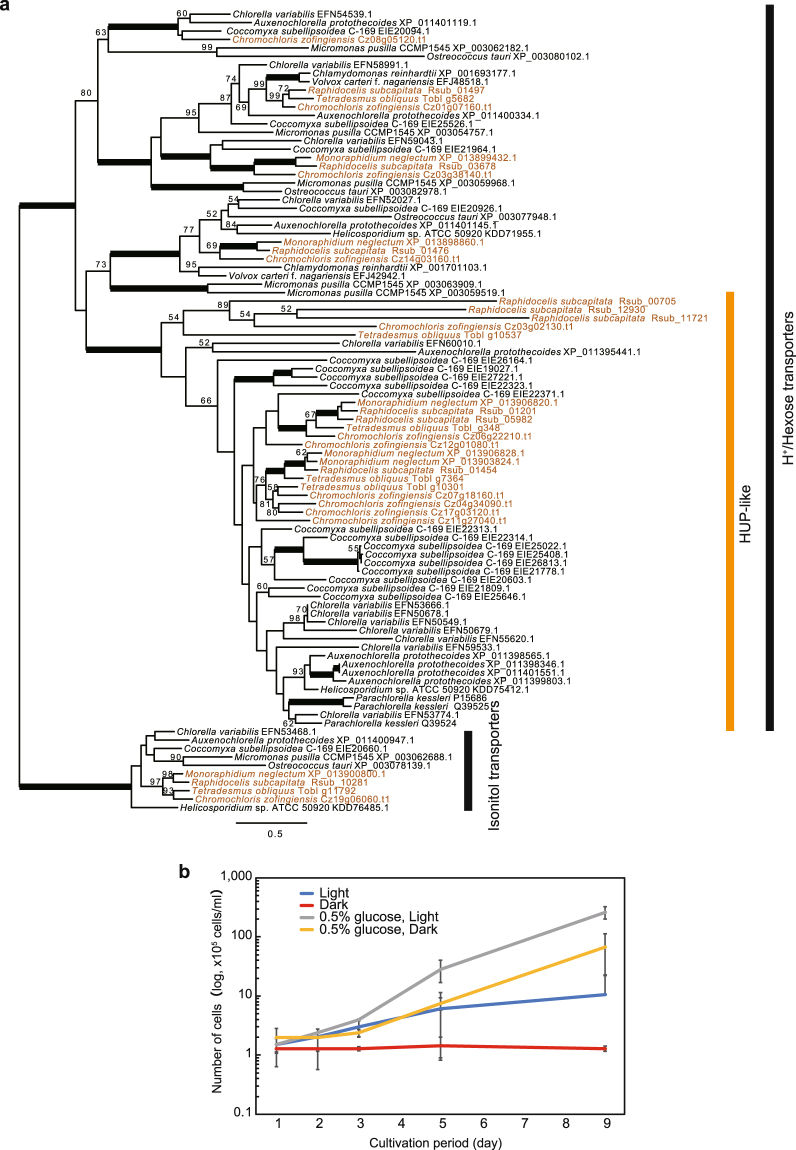
Phylogenetic analysis of H^+^/Hexose cotransporters and growth curves of *Raphidocelis subcapitata*. (**a**) ML tree of H^+^/hexose cotransporters in green algae. Inositol transporters are used as an outgroup. Orange-coloured operational taxonomic units represent proteins of the Sphaeropleales. Bootstrap support (BP) is indicated above the lines where BP is more than 50%. Bold lines represent 100% BP. The ML analysis was performed using IQ-tree 1.5.5 with 348 amino acids with LG + F + G4 model. Non-parametric bootstrapping was performed 100 times. (**b**) Growth curves of *R*. *subcapitata* under autotrophic and heterotrophic conditions. Blue, red, grey, and orange lines represent cultures under light without glucose, continuous dark without glucose, light with glucose, and continuous dark with glucose, respectively. The three independent cultures for each cultivated condition were counted three times. Error bars represent 95% confidence intervals.

The H^+^/hexose cotransporter is functionally related to intake of glucose from the outside of cells^42,43^. A trebouxiophyte, *Parachlorella kessleri*, also has H^+^/hexose cotransporter genes^44^. They are categorized in three classes in green algae, based on the phylogenetic relationships^45^. In the classes, genes in the HUP-like class are increased uniquely in mixotrophic green algae (e.g. *Chlorella* and *Auxenochlorella*), and thus the *HUP-like* is possibly related to the heterotrophic lifestyle^45^. Expression of this gene is induced during heterotrophic growth in *P*. *kessleri*. The mRNA was not detected in photosynthetically-grown cells, but immediately induced by glucose under darkness^46^. Interestingly, our phylogenetic analysis showed that *R*. *subcapitata*, *M*. *neglectum*, *T*. *obliquus*, and *C*. *zofingiensis* had 6, 3, 4, and 7 *HUP-like* genes, respectively, and they were monophyletic with heterotrophic or mixotrophic trebouxiophytes (Fig. 6a). This result suggests that the *HUP-like* genes of the trebouxiophytes and the Sphaeropleales had the same origin, and the genes were acquired in the common ancestor of the trebouxiophytes and the Sphaeropleales but were lacking in the Chlamydomonadales. However, it is also possible, although unlikely, that the gene has been acquired independently in the trebouxiophytes and the Sphaeropleales via horizontal gene transfer and increase in each group. Genome information of the OCC clade is needed to clearly reveal their evolutionary history. Heterotrophic growth using hexose or other simple sugars (e.g. glucose) was observed in *Monoraphidium* sp.^47^, *T*. *obliquus*^48,49^, and *C*. *zofingiensis*^50,51^. To investigate the heterotrophic and mixotrophic growth ability of *R*. *subcapitata*, we cultured it with and without 0.5% glucose under light or continuous dark conditions for 9 days (Fig. 6b; Supplementary Fig. S9). Final cell concentration was the highest in the culture treatment with glucose under light; cell concentration was ~20-fold higher in this treatment than in the treatment without glucose under light. The treatment with glucose under continuous darkness had a ~6-fold higher cell concentration than the treatment without glucose under light; cells could not grow without glucose under continuous darkness. These results suggest that *R*. *subcapitata* is mixotrophic and can grow under heterotrophic conditions without photosynthesis. The cells cultured with glucose contained many lipid bodies (Supplementary Figs S9, S10), suggesting that *R*. *subcapitata* stores excess glucose as TAG in these lipid bodies. In the case of nitrogen resources, we identified a large number of amino acid/peptide transporter genes in the Sphaeropleales, although the number of nitrate/nitrite transporter genes was not different among the Sphaeropleales and *C*. *reinhardtii*. In *Chlorella vulgaris*, glucose or glucose analogues induce the intake of amino acids through amino acid permeases^52^, even if inorganic nitrogen (i.e. ammonium or nitrate) is present in high amounts^53^. Based on these findings, it appears reasonable that the Sphaeropleales possess a large number of H^+^/hexose cotransporters and amino acid permeases. These transporters may contribute to their rapid growth under different nutrient conditions.

*M*. *neglectum* and *T*. *obliquus* can grow under highly salinity conditions (up to 1% NaCl)^10,54^. The adaptation to high salt stress may be conferred by the large number of genes for aquaporin (Table 2). Aquaporin can passively transport small polar molecules, such as water, across cell membranes in different species of algae^55^, and is likely to be used for controlling intracellular osmotic pressure. Multiple metal-nicotianamine transporters may be related to high sensitivity of the Sphaeropleales to exogenous metals. Metal-nicotianamine transporters transport various metals, such as Fe(III), Fe(II), Ni(II), Zn(II), Cu(II), Mn(II), and Cd(II) as metal-PS (phytosiderophores) or metal-NA (nicotianamine) chelates in *Zea mays*^56,57^. In *Scenedesmus*, organic chelators are released in inorganic medium^58^, and uptake of iron is regulated by siderophore secretion^59^. These findings suggest that the large number of genes for metal-nicotianamine transporters in *R*. *subcapitata* may induce high sensitivity to different metals. *M*. *neglectum*, *T*. *obliquus*, and *C*. *zofingiensis* also possessed multiple copies of genes for metal-nicotianamine transporters, suggesting that the Sphaeropleales might have high sensitivity to metals. In our homology search, there were few genes for metal-nicotianamine transporters among green algae, except for the Sphaeropleales; however, their origins are unknown because of their sequence diversity. The increase in transporters in the Sphaeropleales is likely to be related to their lifestyle. In the Sphaeropleales cell cycle, the stage with motility (e.g. flagellates) is not dominant or not known^60,61^. Immobility may force cells to adapt to different environmental conditions aided by their numerous transporters.

## Conclusions

The Sphaeropleales are a dominant group of green algae and contain species important in ecosystems and those with potential for applied usage. In this study, we sequenced the nuclear, plastid, and mitochondrial genomes of *R*. *subcapitata*, and performed comparative analyses of Sphaeropleales species. The plastid and mitochondrial genomes provided insights into the phylogenetic relationships of *R*. *subcapitata* and the complex evolutionary histories in the Sphaeropleales. The nuclear genome of *R*. *subcapitata* was the more compact genome (i.e. assembly size) than those of *M*. *neglectum*, *T*. *obliquus*, and *C*. *zofingiensis*. The gene repertoire was conserved in Sphaeropleales species. Comparison of transporter genes indicated that the Sphaeropleales have the potential to adapt to different natural environmental conditions. These findings have implications for future ecological research and applications such as biomarkers and screening of highly oleaginous algae.

## Methods

### Culture

*R*. *subcapitata* NIES-35 (=ATCC22662) is a strain widely used in environmental bioassays e.g.^60^. It is an axenic strain that is available from the Microbial Culture Collection at the National Institute for Environmental Studies, Japan (http://mcc.nies.go.jp). The strain was cultivated at 20 °C in C medium^62^ under white LED (~20 µmol photons/m^2^/s) with 12 h:12 h light:dark cycles in 300 mL glass flasks.

For tests of cultivation under mixotrophic or heterotrophic conditions, the cells were cultivated in 6 mL of C medium or C medium with 0.5% glucose in glass test tubes under the above light conditions or continuous darkness. Three separate *R*. *subcapitata* cultures were grown in each type of medium and light conditions. The cells were shaken using a TAITEC small size shaker NR-3 (TAITEC, Tokyo, Japan) at ~150 rotations per minute. The initial cell concentration was approximately 1.7 × 10^5^ cells/mL. Cell concentrations were counted three times using a haemocytometer.

### DNA and RNA extraction

For DNA extraction, *R*. *subcapitata* was cultured for 2 weeks in 500 mL of C medium. Cells were collected by gentle centrifugation and ground in a pre-cooled mortar with liquid nitrogen and 50 mg of 0.1 mm glass beads (Bertin, Rockville, MD, USA). The cells were incubated with 600 µL of CTAB extraction buffer^63^ at 65 °C for 1 h. DNA was separated by mixing with 500 µL of chloroform and centrifuging at 20,000 × *g* for 1 min; it was concentrated by standard EtOH precipitation. For RNA sequencing, cells were cultured for a week in 100 mL C medium. To acquire whole expressed genes, RNA was extracted twice just before light- and dark-phase. Cells were collected and ground as described above, and RNA was extracted using the RNeasy Mini Kit (Qiagen, Hilden, Germany). DNA contamination was removed using the TURBO DNA-free Kit (Thermo Fisher Scientific, Waltham, MA, USA). Equal amounts of light- and dark-RNA samples were mixed and sequenced at the same time.

### Sequencing

For DNA libraries, we prepared paired-end (~550 bp insert) and mate-pair (~3–4 kbp insert) libraries. The paired-end library was prepared using the TruSeq Nano DNA Library Prep Kit for NeoPrep (Illumina, San Diego, CA, USA) with the NeoPrep system (Illumina) following the manufacturer’s protocol. The mate-pair library was prepared using the Nextera Mate Pair Sample Preparation Kit (Illumina) following the manufacturer’s protocol. We also prepared a paired-end RNA library (~550 bp insert) using the NEBNext Ultra Directional RNA Library Prep Kit for Illumina (New England BioLabs, Inc., Ipswich, MA, USA). mRNA was purified using the NEBNext Poly(A) mRNA Magnetic Isolation Module (New England BioLabs). All libraries were sequenced on the MiSeq sequencing system (Illumina) using the MiSeq Reagent Kit v3, 600 cycles (300 bp × 2), and the MiSeq Reagent Kit v2, 150 cycles (75 bp × 2), for the paired-end and mate-pair libraries, respectively.

### Assembly and annotation

We acquired 3,847,746, 16,768,250, and 1,394,371 read pairs for DNA paired-end, mate-pair, and RNA paired-end libraries, respectively. The reads were deposited in DDBJ/Genbank/ENA with accession numbers DRR090198 (DNA paired-end), DRR090199 (DNA mate-pair), and DRR090200 (RNA paired-end). The sequences were trimmed using Trimmomatic 0.36^64^ with default options. The DNA paired-end reads were used for *in silico* genome size estimation by Jellyfish 2.2.6^65^ with the 17-mer option. The genome was assembled using SPAdes Genome Assembler 3.9.0^66^ with default options. Scaffolding was performed using SSPACE-standard 3.0^67^, and some gaps were closed by GapFiller v1.10^68^. To extract plastid and mitochondrial sequences, we performed a blastx^69^ search using available plastid and mitochondrial proteins of the Sphaeropleales as a query. The plastid and mitochondrial genomes were automatically annotated using Prokka v1.11^70^, and manually curated on an Artemis genome browser^71^. rRNA and tRNA were also searched using RNAmmer 1.2^72^ and tRNAscan-SE 2.0^73^, respectively. Group I and II introns were initially detected using RNAweasel^74^, and curated by alignments with homologs. Tandem repeats were found using Tandem Repeats Finder 4.09^75^. Gene models of the nuclear scaffolds were constructed following the funannotate pipeline 0.3.7 (https://github.com/nextgenusfs/funannotate). To acquire evidence for gene model construction, we mapped RNA-seq reads to the scaffolds using HISAT2 2.0.4^76^, and constructed transcript-based gene models using Trinity 2.2.0^77^ and PASA pipeline 2.0.2^78^. For the nuclear genome of *T*. *obliquus*, because annotation was not available, we annotated the scaffolds (accession number GCA_900108755.1). Repeat regions of the scaffolds were soft-masked using RepeatModeler and RepeatMasker (http://www.repeatmasker.org). Gene models were predicted using AUGUSTUS^79^, for which training was performed using BUSCO^28^ with the eukaryote dataset. The nuclear, plastid, and mitochondrial genomes of *R*. *subcapitata* were deposited in DDBJ/Genbank/ENA with accession numbers BDRX01000001–BDRX01000300, AP018038, and AP018037, respectively.

### Phylogenetic analyses

To infer the phylogenetic relationships between *R*. *subcapitata* and other species of the Sphaeropleales, we performed phylogenetic analyses using plastid and mitochondrial genome-encoded proteins. For plastid proteins, we used 55 proteins in the dataset described by Fučíková *et al.*^20^ (AtpA, AptB, AtpE, AtpF, AtpH, AtpI, CcsA, CemA, ClpP, PetB, PetD, PetG, PetL, PsaA, PsaB, PsaC, PsaJ, PsbA, PsbB, PsbC, PsbD, PsbE, PsbF, PsbI, PsbJ, PsbK, PsbL, PsbM, PsbN, PsbT, PsbZ, RbcL, Rpl2, Rpl5, Rpl14, Rpl16, Rpl20, Rpl23, Rpl36, RpoA, RpoBa, RpoBb, RpoC2, Rps3, Rps4, Rps7, Rps8, Rps9, Rps11, Rps12, Rps14, Rps18, Rps19, TufA, and Ycf3). The dataset was composed of 12 organisms in the Sphaeropleales. The complete plastid genome of *Ourococcus multisporus* was unavailable, but instead, we used its protein sequences. *Mychonastes homosphaera* was excluded from the dataset because of its rapid evolutionary rate. *D*. *salina* and *C*. *reinhardtii* were used as an outgroup. For the mitochondrial dataset, we used all mitochondrial genome encoding proteins: Atp6, Atp9, Cob, Cox1, Cox2a, Cox3, Nad1, Nad2, Nad3, Nad4, Nad4L, Nad5, and Nad6. The dataset was composed of 17 organisms. *Stigeoclonium helveticum* (Chaetophorales) was used as an outgroup. Both sequences were aligned using MAFFT v7.221^80^, and highly divergent regions were manually trimmed with MEGA 6.0.6^81^. Substitution models were tested using IQ-TREE 1.4.4^82^. Maximum likelihood (ML) analyses were performed using RAxML 8.2.9^83^ with the LG + GAMMA + F + I model. Non-parametric bootstrap analyses were replicated 200 times. Bayesian analyses were performed using MrBayes v3.2.6^84^ with the same substitution model. The inference consisted of 1,000,000 generations with sampling every 1,000 generations using four Metropolis-coupled Markov chain Monte Carlo (MCMCMC) simulations. Two separate runs were performed, and Bayesian posterior probabilities were calculated from the majority rule consensus of the tree sampled after the initial 250 burn-in trees.

### Identification of gene families, NUMTs, and genes for lipid biosynthesis and transporters

Gene families were searched using TreeFam 9^29^ with an e-value cut-off of <1E^−5^. NUMTs were searched by blastn with the mitochondrial genomes as a query, with an e-value cut-off of <1E^−3^. Genes for lipid biosynthesis were based on Bogen *et al.*^10^, and searched using blastp with the proteins of *Arabidopsis thaliana* as a query, with an e-value cut-off of <1E^−10^. Transporters were identified using blastp with the Transporter Classification Database (TCDB)^85^ as a query, with an e-value cut-off of <1E^−5^.

### Data availability

The strain used in this study (*R*. *subcapitata*, NIES-35) is available from the Microbial Culture Collection at the National Institute for Environmental Studies (NIES) (http://mcc.nies.go.jp), Japan. The DNA paired-end, mate-pair, and RNA paired-end reads were deposited in DDBJ/Genbank/ENA with accession numbers DRR090198, DRR090199, and DRR090200, respectively. The nuclear, plastid, and mitochondrial genomes were deposited in DDBJ/Genbank/ENA with accession numbers BDRX01000001–BDRX01000300, AP018038, and AP018037, respectively.

## Electronic supplementary material

Supplementary figures

Supplementary tables
