# Supplementary material for: Raphidocelis subcapitata (=Pseudokirchneriella subcapitata) provides an insight into genome evolution and environmental adaptations in the Sphaeropleales
Source: Sci Rep. 2018 May 23;8:8058. doi: 10.1038/s41598-018-26331-6 (PMC5966456; doi:10.1038/s41598-018-26331-6)
Supplement: Supplementary file 1 — Supplementary figures [file 41598_2018_26331_MOESM1_ESM.pdf]

Supplementary Information

Raphidocelis subcapitata **(=*Pseudokirchneriella subcapitata*) provides an insight into genome evolution and environmental adaptations in the Sphaeropleales**

Shigekatsu Suzuki*, Haruyo Yamaguchi, Nobuyoshi Nakajima, and Masanobu Kawachi

Center for Environmental Biology and Ecosystem Studies, National Institute for Environmental Studies, Ibaraki, Japan

*Author for correspondence: Center for Environmental Biology and Ecosystem Studies, National Institute for Environmental Studies, Ibaraki, Japan, Phone: +81 29 850 2204, Fax: +81 29 850 2587

Email: suzuki.shigekatsu@nies.go.jp

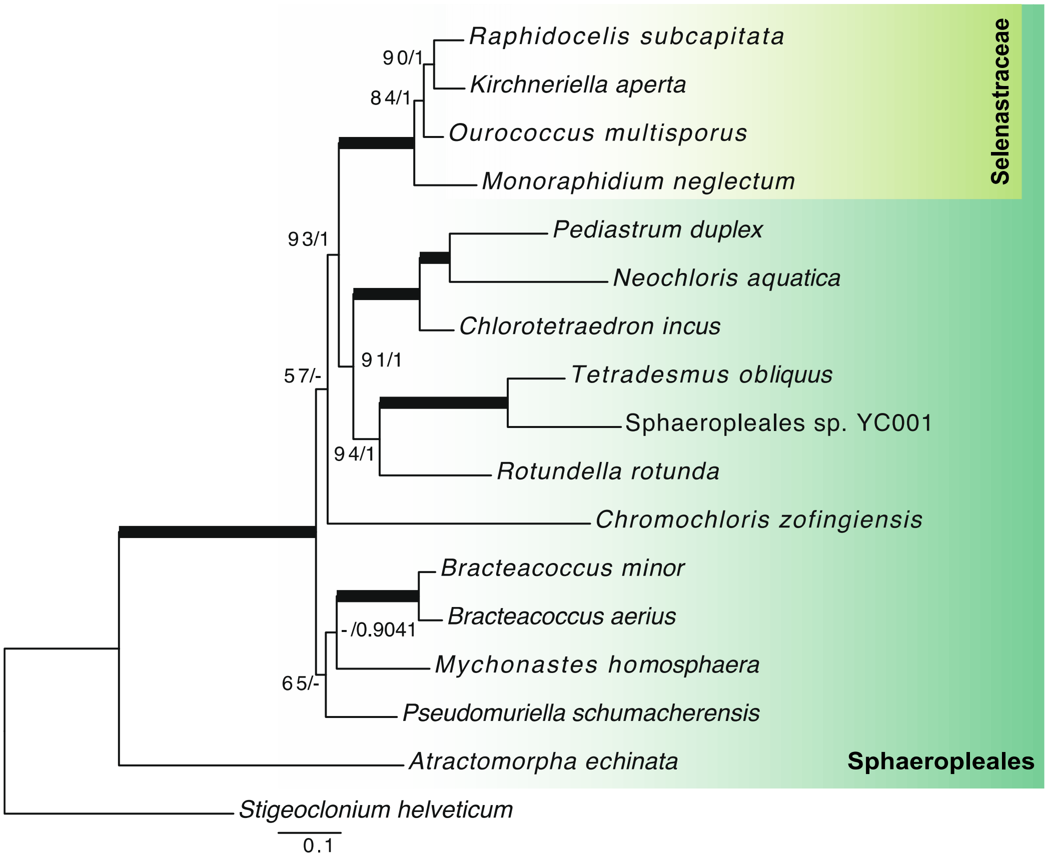

Supplementary Figure S1. ML phylogenetic tree of the Sphaeropleales using 13 mitochondrion-encoding proteins. The best tree was reconstructed using a concatenated dataset of 3,144 amino acids. Values at the nodes represent bootstrap supports (BP) of 200 replicates (right) and Bayesian posterior probabilities (BPP) (left). BP < 50 or BPP < 0.9 are not shown. Bold lines represent BP = 100 and BPP = 1.00.

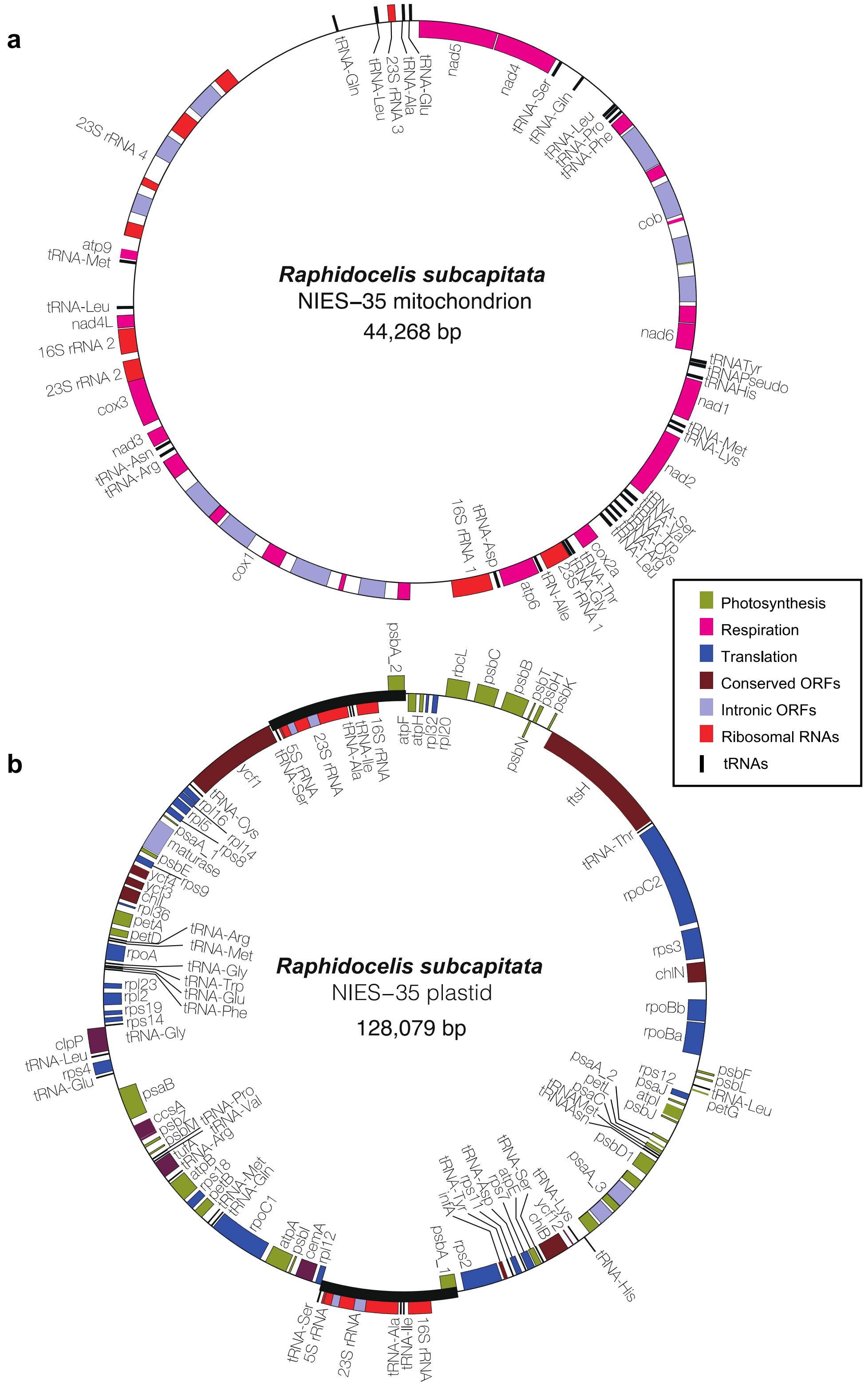

Supplementary Figure S2. Genome maps of mitochondrial and plastid genomes of *Raphidocelis subcapitata.*

Gene maps of the mitochondrial (a) and plastid (b) genomes of *R. subcapitata*. Genes are shown in different colours according to their putative functions. Genes on the outside of the maps are transcribed in a clockwise direction, and genes inside the maps are transcribed in a counter-clockwise direction.

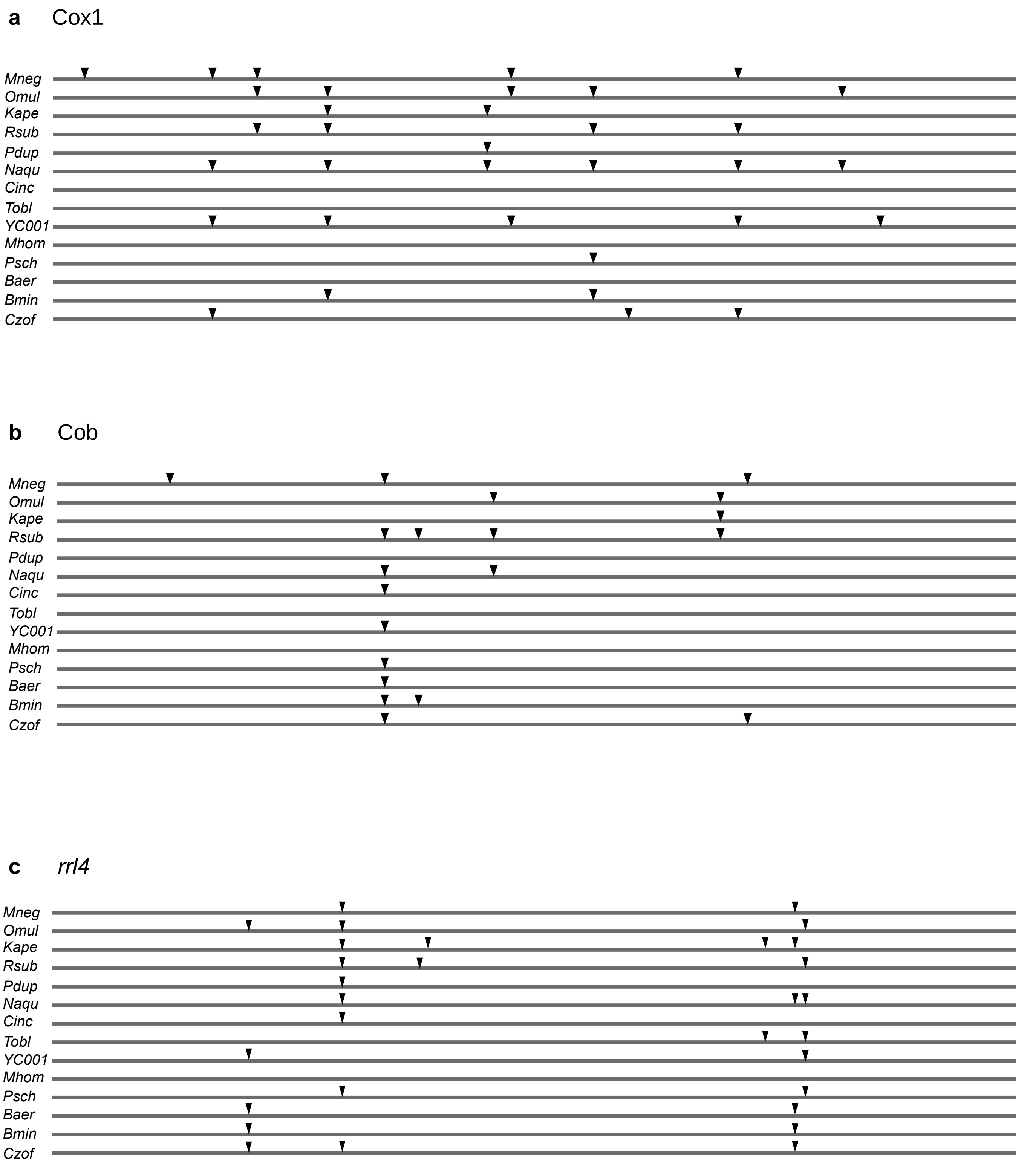

Supplementary Figure S3. Intron-inserted positions of genes in mitochondrial genomes of the Sphaeropleales.

Intron-inserted positions of *Cox1* (a), *Cob* (b), and *rrl4* (c) are shown. Arrowheads represent introns.

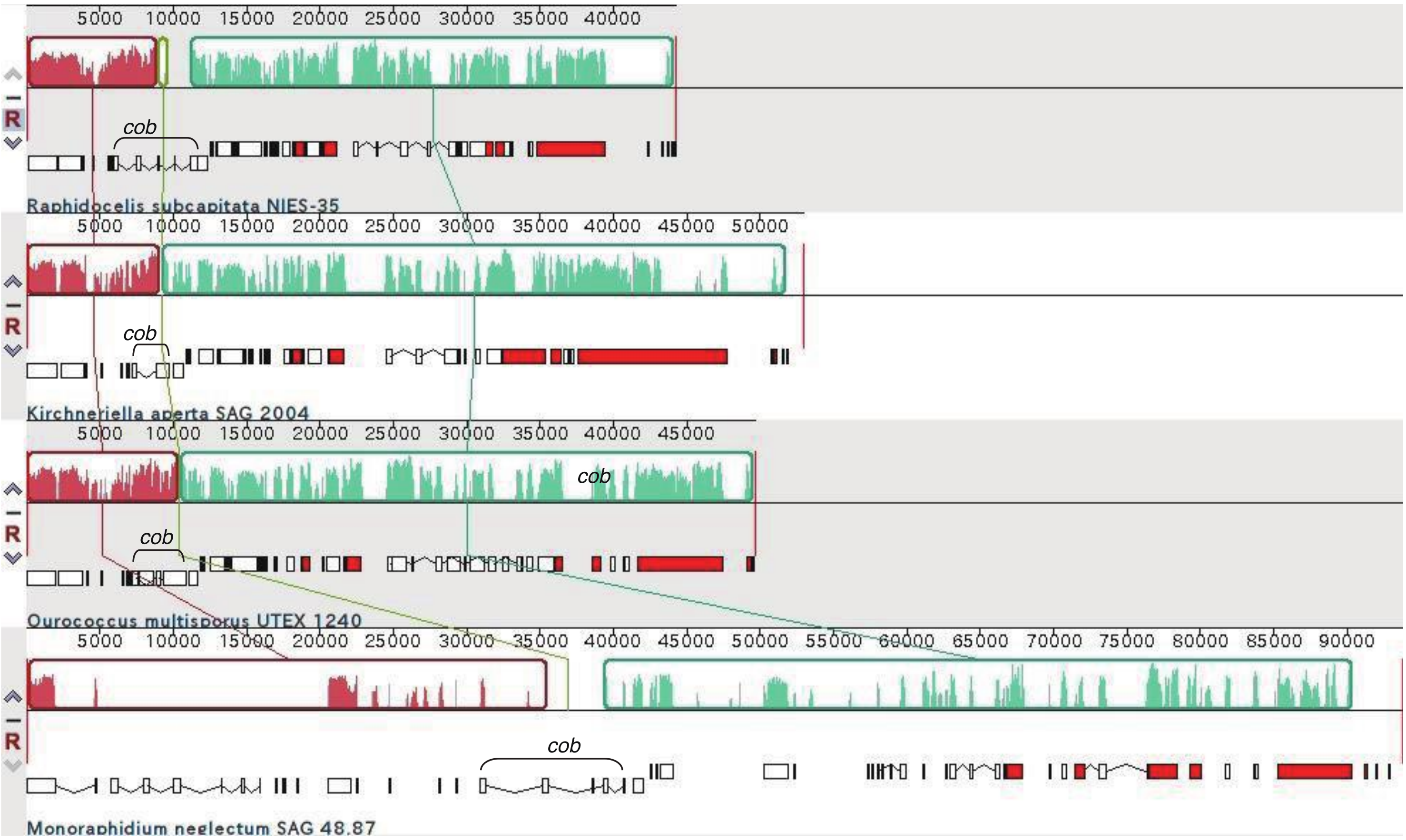

Supplementary Figure S4. ProgressiveMauve alignments of mitochondrial genomes of *Raphidocelis subcapitata*, *Kirchneriella aperta*, *Ourococcus multisporus*, and *Monoraphidium neglectum*.

Coloured bars in syntenic blocks represent similarities between the genomes.

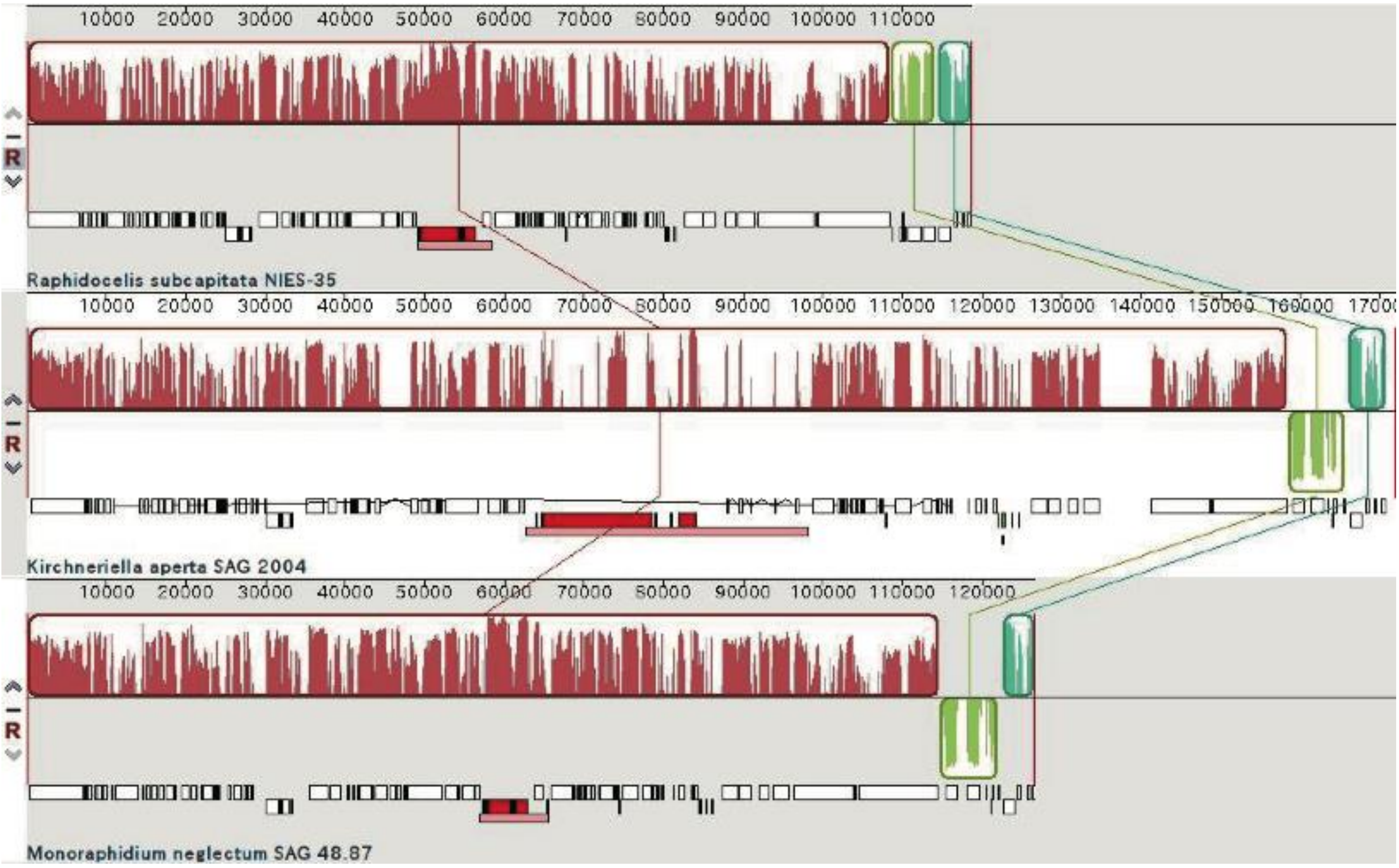

Supplementary Figure S5. ProgressiveMauve alignments of plastid genomes of *Raphidocelis subcapitata*, *Kirchneriella aperta*, and *Monoraphidium neglectum*.

Coloured bars in syntenic blocks represent similarities between genomes. The one inverted repeat is omitted from this analysis.

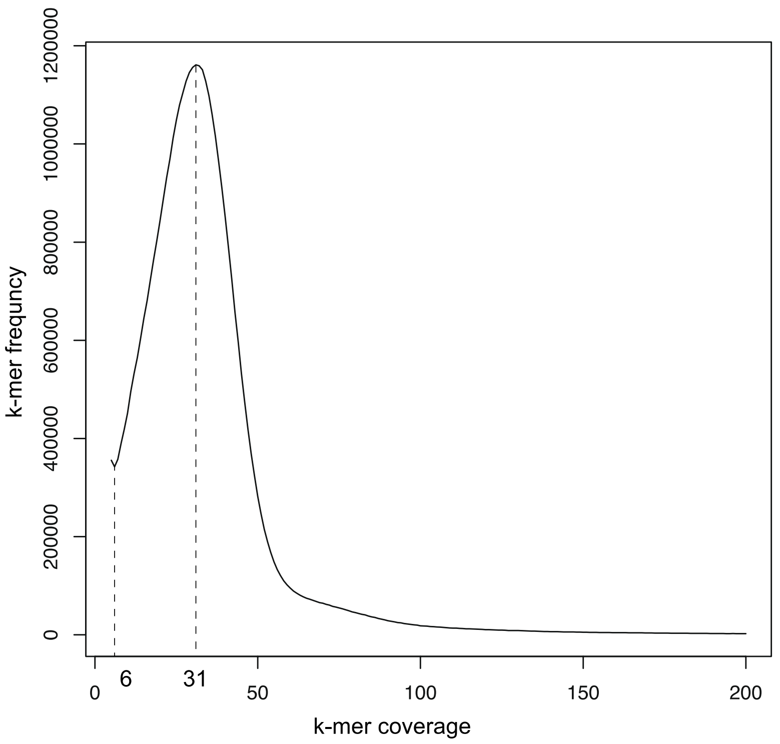

Supplementary Figure S6. *In silico* estimation of the genome size of *R. subcapitata*.

The k-mer (17-mer) coverage is peaked at 31. The predicted genome size is 46,790,751 bp.

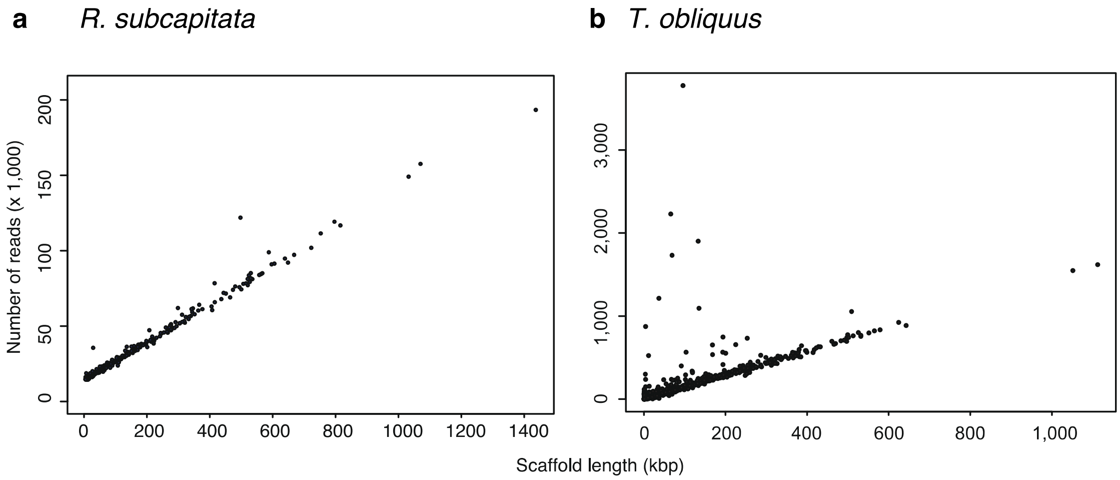

Supplementary Figure S7. Plots of scaffold length vs. number of mapped reads of *Raphidocelis subcapitata* and *Tetradesmus obliquus*.

The plots of *R. subcapitata* (a), and *T. obliquus* (b). The reads were mapped on the scaffolds. The plots are on one line, suggesting that the genomes have haploid characters.

```
                                    1
Volvox_carteri_f._nagariensis       MA--DAKEQL KAKLKADPSF RAELKDRIKG ALLSKVPAT- -QAISYNFDS
Chlamydomonas_incerta               MF--ESKDHL KQKLKADPSF RAELKDRIKS ALLSKVPAS- -VPISYNFDS
Chlamydomonas_reinhardtii           MS--ESKDQL KEKLKADPSF RAELKDRIKN ALLSKVPAS- -VPISYNFDS
Gonium_pectorale                    MT--DAKDAL KQQLKQNPSF RAELKERIKS ALVAKVPAP- -QLISYNFDS
Polytomella_sp._Pringsheim_198.80   MS--DAKDQL KEQLKASPSF RAELKDKLKA ALLSKVPAS- -QPIQYNFDS
Raphidocelis_subcapitata            MVD-SAKEAI KAKLKADPNF RAELRDRVKA ALLAKANGEG LKTAEYSFDS
Monoraphidium_neglectum             T---AAAAAA TAAAGGSGTL RAELRERVKT ALLEKAAGEG LKTAEYNFDS
Tetradesmus_obliquus_cox2b1         MVDQSAREAL KAKLKADPSF RKELRKRVTD SLLAKSDED- PKTAQYNFDA
Tetradesmus_obliquus_cox2b2         MVDQSAREAL KAKLKADPSF RKELRKRVTD SLLAKSDED- PKTAQYNFDA
Chromochloris_zofingiensis          M---DLRPEL KEKLRSDPEF KREVRDRIKA ALLDKVKET- -KKAEYQFDS

                                   51
Volvox_carteri_f._nagariensis       YMLTE----- ---------- ---------- ---------- ----VQPGQL
Chlamydomonas_incerta               YMLTE----- ---------- ---------- ---------- ----VQPGQL
Chlamydomonas_reinhardtii           YMLTE----- ---------- ---------- ---------- ----VQPGQL
Gonium_pectorale                    YMLTE----- ---------- ---------- ---------- ----VQPGQL
Polytomella_sp._Pringsheim_198.80   YMVTD----- ---------- ---------- ---------- ----VQPGQL
Raphidocelis_subcapitata            YMLTD----- ---------- ---------- ---------- ----PEPGQL
Monoraphidium_neglectum             YMLTD----- ---------- ---------- ---------- ----PEPGQL
Tetradesmus_obliquus_cox2b1         YMLTD----- ---------- ---------- ---------- ----VEPGQI
Tetradesmus_obliquus_cox2b2         YMLTD----- ---------- ---------- ---------- ----VEPGQI
Chromochloris_zofingiensis          YMLSGKSKAA VSALVLFYTL DRQSGVPCNL DLLINVPSSI ILSYVEPGQL

                                  101
Volvox_carteri_f._nagariensis       RVLEVDERLV LPTNTLIRLL VTASDVLHSW AVPALGVKMD AIPGRLNQVW
Chlamydomonas_incerta               RVLEVDERLV LPTNTLIRLL VTASDVLHSW AVPALGVKMD AVPGRLNQVW
Chlamydomonas_reinhardtii           RVLEVDERLV LPTNTLIRLL VTASDVLHSW AVPALGVKMD AVPGRLNQVW
Gonium_pectorale                    RVLEVDERLV LPTNTLVRLL VTASDVIHSW AVPALGVKMD AVPGRLNQVW
Polytomella_sp._Pringsheim_198.80   RMLEVDERLV LPTNTLVRLL VTASDVIHSW AVPSLGIKMD AIPGRLNQIW
Raphidocelis_subcapitata            RLLDVDERLV LPTNSLIRLL VTSTDVIHSW AVPSLGIKID AVPGRLNQVW
Monoraphidium_neglectum             RLLDVDERLV LPTNSLIRVL VTSSDVIHSW AVPSLGVKID AIPGRLNQVC
Tetradesmus_obliquus_cox2b1         RLLDCDERLV LPTNSLVRLL VTSSDVIHSW AVPSLGVKID AIPGRLNQVW
Tetradesmus_obliquus_cox2b2         RLLDCDERLV LPTNSLVRLL VTSSDVIHSW AVPSLGVKID AIPGRLNQVW
Chromochloris_zofingiensis          RLLDVDERLV LPTNSLIRVL VTSADVIHSW AVPSLGVKVD AVPGRLNQVW

                                  151
Volvox_carteri_f._nagariensis       LSINREGVFY GQCSELCGAN HSFMPIVVEA ISPRQFLTEY VKKWI-----
Chlamydomonas_incerta               MSINREGVFY GQCSELCGAN HSFMPIVVEA ISPRQFLTEY VKKWI-----
Chlamydomonas_reinhardtii           MSINREGVFY GQCSELCGAN HSFMPIVVEA ISPRQFLTEY VKKWI-----
Gonium_pectorale                    LSINREGVFY GQCSELCGAN HTFMPIVVEA ISPRQFLTEY VKKWI-----
Polytomella_sp._Pringsheim_198.80   LTINREGVFY GQCSEICGAN HSFMPIVVEA ISPRAFLTEY VKKWI-----
Raphidocelis_subcapitata            LTIQRPGVFY GQCSELCGAN HSFMPIVVEA VTPRQWIQQY LPKWM-----
Monoraphidium_neglectum             VLL------- ---------- ---LP----- ---------- ----------
Tetradesmus_obliquus_cox2b1         ITIQRPGVFY GQCSELCGAN HAFMPIVVEA VTPRQFVQVI VPLIVTVVDL
Tetradesmus_obliquus_cox2b2         ITIQRPGVFY GQCSELCGAN HAFMPIVVEA VTPRQFVQN- GKTMVDGLDA
Chromochloris_zofingiensis          VTIQRPGVFY GQCSELCGAN HAFMPICVEA VTPRQYVTEY LKKWM-----

                                  201
Volvox_carteri_f._nagariensis       --S
Chlamydomonas_incerta               --S
Chlamydomonas_reinhardtii           --S
Gonium_pectorale                    --S
Polytomella_sp._Pringsheim_198.80   --Q
Raphidocelis_subcapitata            --S
Monoraphidium_neglectum             --Q
Tetradesmus_obliquus_cox2b1         KEQ
Tetradesmus_obliquus_cox2b2         GNG
Chromochloris_zofingiensis          --E
```

Supplementary Figure S8. Alignment of *cox2b* of the Chlamydomonadales and the Sphaeropleales.

Additional N-termini are indicated by a red square.

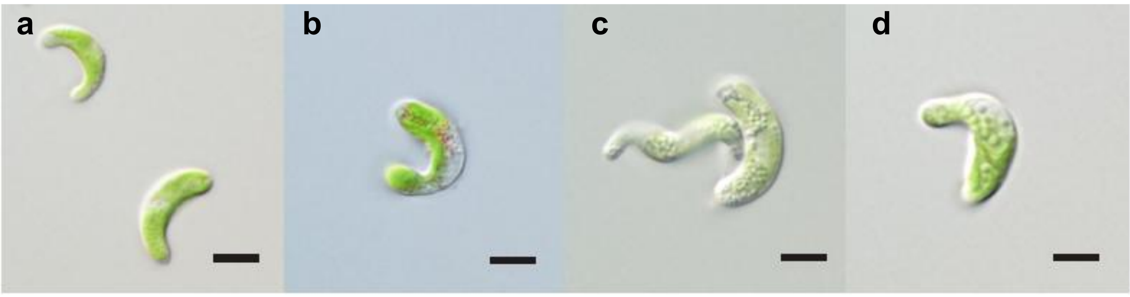

Supplementary Figure S9. Light microscopic images of *Raphidocelis subcapitata* under autotrophic, mixotrophic, and heterotrophic conditions.

Light microscopic images of *R. subcapitata* cultured for nine days under light without glucose (a), continuous darkness without glucose (b), light with 0.5% glucose (c), and continuous darkness with 0.5% glucose (d). Scale bars represent 5 µm.

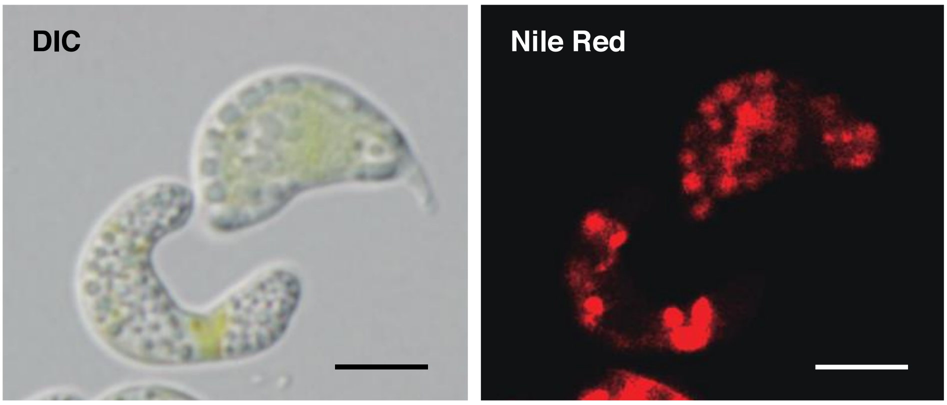

Supplementary Figure S10. Nile red staining of *Raphidocelis subcapitata*.

Cells were cultivated in C medium with 0.5% glucose under light. They were stained with 1 µg/mL Nile red. Scale bars represent 5 µm.
